# Physical distancing messages targeting youth on the social media accounts of Canadian public health entities and the use of behavioral change techniques

**DOI:** 10.1186/s12889-021-11659-y

**Published:** 2021-09-07

**Authors:** Sheryll Dimanlig-Cruz, Arum Han, Samantha Lancione, Omar Dewidar, Irina Podinic, OPH-SEPH Collaborative (OSC) Working Group, Baies Haqani, Justin Haug, Lynne Leonard, Elaine Medline, Andrea Patey, Justin Presseau, Emily Thompson, Monique Potvin Kent, Melissa Brouwers

**Affiliations:** 1 School of Epidemiology and Public Health (SEPH), Faculty of Medicine, University of Ottawa, Ottawa, Canada; 2 Ottawa Public Health (OPH), Ottawa, Ontario Canada

**Keywords:** SARS-CoV-2, COVID-19, Social media, Physical distancing, Social distancing, Youth, Young adult, Behavioral change techniques

## Abstract

**Introduction:**

Physical distancing (PD) is an important public health strategy to reduce the transmission of COVID-19 and has been promoted by public health authorities through social media. Although youth have a tendency to engage in high-risk behaviors that could facilitate COVID-19 transmission, there is limited research on the characteristics of PD messaging targeting this population on social media platforms with which youth frequently engage. This study examined social media posts created by Canadian public health entities (PHEs) with PD messaging aimed at youth and young adults aged 16–29 years and reported behavioral change techniques (BCTs) used in these posts.

**Methods:**

A content analysis of all social media posts of Canadian PHEs from Facebook, Twitter, Instagram and YouTube were conducted from April 1st to May 31st, 2020. Posts were classified as either implicitly or explicitly targeting youth and young adults. BCTs in social media posts were identified and classified based on Behavior Change Technique Taxonomy version 1 (BCTTv1). Frequency counts and proportions were used to describe the data.

**Results:**

In total, 319 youth-targeted PD posts were identified. Over 43% of the posts originated from Ontario Regional public health units, and 36.4 and 32.6% of them were extracted from Twitter and Facebook, respectively. Only 5.3% of the total posts explicitly targeted youth. Explicit posts were most frequent from federal PHEs and posted on YouTube. Implicit posts elicited more interactions than explicit posts regardless of jurisdiction level or social media format. Three-quarters of the posts contained at least one BCT, with a greater portion of BCTs found within implicit posts (75%) than explicit posts (52.9%). The most common BCTs from explicit posts were i*nstructions on how to perform a behavior* (25.0%) and *restructuring the social environment* (18.8%).

**Conclusions:**

There is a need for more PD messaging that explicitly targets youth. BCTs should be used when designing posts to deliver public health messages and social media platforms should be selected depending on the target population.

**Supplementary Information:**

The online version contains supplementary material available at 10.1186/s12889-021-11659-y.

## Background

In early January 2020, the World Health Organization (WHO) released a statement forewarning of a new type of coronavirus, SARS-CoV-2, better known as coronavirus disease 2019 (COVID-19), as a cluster of patients with unusual pneumonia-like symptoms were first detected in Wuhan, China [1–3]. With limited information about treatment and prevention, governments and health organizations worldwide have enforced physical (or social) distancing measures in addition to hand hygiene, quarantine, and contact tracing as primary means to limit the spread of SARS-CoV-2 and mitigate the impact of the outbreak. Although COVID-19 vaccines have been developed and approved as of late 2020, the importance of non-pharmaceutical interventions, including physical distancing (PD), remain in order for effective vaccine rollout [4]. For most jurisdictions, PD measures include maintaining at least 2 m or 6 ft distance between people, restricting face-to-face interactions with people who are not from the same household (or social bubble), prohibiting large gatherings, and limiting activities outside the home. The effectiveness of these non-pharmacologic interventions to ‘flatten the curve’ relies on the strict compliance of individuals [5–7].

Unfortunately, certain individuals may be less willing to comply with these PD measures. After a national emergency was declared in response to the COVID-19 pandemic, an American study found that 68.6% of youth between the ages of 13 and 18 did not practice PD because they thought that they had a low risk of infection and an even lower risk for death [8]. Several other international studies have also reported that compliance rates for PD are the lowest for people aged 30 years and under [9, 10]. Indeed, youth and young adults (commonly defined as individuals between the ages of 10 and 24 years) are progressing through critical developmental periods that are associated with increased autonomy, susceptibility to peer influence, risk-taking behaviours and importance of social connections [11]. As a result, they may experience greater difficulty adhering to strict public health measures implemented in response to COVID-19.

Researchers have suggested that public health campaigns addressing COVID-19 health and safety measures should focus on youth and young adults to promote compliance with PD and emphasize the contribution of the individual’s behavior against community transmission [9, 12–14]. Social media platforms, often used by youth, have been utilized to deliver large-scale public health interventions targeting youth health behaviors such as smoking, sexual health and mental health. These campaigns have had a variety of successes on participation and engagement and limited information on individual behavior change [15–19]. Social media offers a leveraged advantage compared to traditional mass media (television, radio and print advertisements) due to its broader reach and personalized marketing capabilities. Each platform allows users to interact with individual posts as well as other users; thus, the potential to influence the public’s behavior, disseminate health information and increase awareness during the COVID-19 pandemic has become attractive to many public health authorities.

Tailored and theoretically driven “behavioral” messaging integrated into content may further improve the effectiveness of social media platforms and serve as an intervention to promote behavior change in health. Interventions to change behaviors are often delivered with a number of behavioral change techniques (BCTs) that are observable, replicable and designed to alter or redirect causal processes to regulate behaviors. They are often referred to as the “active ingredient” within an intervention [20]. Based on a comprehensive study by Web et al. [21], internet-based interventions with theoretical bases had a greater impact in change of health-related behaviors compared to those with none, and similar results were seen among interventions with more BCTs than interventions with lesser incorporated techniques [21, 22]. In the context of the COVID-19 pandemic, several researchers expressed the need for effective interventions with insights and evidence from behavioral science given the urgency of the current situation [23–25].

The objectives of this study were to: (1) to assess the content and format of PD messaging targeted at youth and young adults aged 16–29 years across social media platforms on accounts belonging to Canadian public health entities (PHEs); and (2) to examine the use of BCTs for PD-related social media posts that implicitly and explicitly target youth.

## Methods

### Context/collaboration

This study stems from a collaborative research project between Ottawa Public Health (OPH) and the University of Ottawa’s School of Epidemiology and Public Health (uOttawa-SEPH), the OPH-SEPH Collaborative (OSC) Group. The OSC was developed in direct response to the COVID-19 outbreak [26]. Using an integrated knowledge translation strategy, the OSC conducted an applied health research project prioritized by OPH focused on PD messaging campaigns targeting youth and young adults by international, national and local PHEs. The goal was to examine how PHEs across different Canadian jurisdictions are considering youth and young adults in their PD-related messaging to establish recommendations for producing effective public health communications for this age group during the COVID-19 pandemic.

### Social media post search strategy and selection criteria

This cross-sectional study involved collecting and analyzing posts from popular social media platforms published by Canadian PHEs that targeted youth, defined by OPH as individuals between the ages of 16 and 29.

#### Public health entities

Additional file 1 shows the overview of Canadian PHEs examined in this study; there were 3 PHEs at the federal level (including Chief Public Health Officer of Canada), 26 at the provincial/territorial level and 34 public health units at the Ontario regional level. Additional file 2 provides the list of PHEs, by level, with their respective social media accounts and metrics listed.

#### Eligible platforms

The official websites of each Canadian PHE were searched for the names and links to their official social media accounts. The most common platforms used by the PHEs were Facebook, Instagram, Twitter, and YouTube. An assessment was then conducted of all accounts across all four platforms.

#### Time interval

Social media posts that were posted from April 1st to May 31st, 2020 inclusive were extracted between June and July 2020. A content analysis of each post was conducted in order to identify all posts related to PD.

### Post selection criteria

#### Eligible posts

Posts were eligible if they were specific to physical and/or social distancing (PD) messaging and identified to be targeted for youth and young adults using the following criteria: (1) specific mention of the terms “youth,” “teen,” or “young adult,” “secondary students,” “post-secondary students,” or an age or age range within 16–29 years of age; (2) featuring actors or characters that look in this age range; (3) mention of themes relevant to this age group (e.g., dating, sexual activity, going to college or university, living with roommates, etc.); (4) use of humour; and (5) offering a fun or innovative way to practice PD that may appeal to youth. To ensure posts were only captured once, only social media posts that were originally created and posted by the PHEs were extracted; any ‘retweets’ or ‘shares’ were excluded in the study. The requirements and definitions of implicitly targeting posts were developed by members of the research team who belong to the target age group. These were subsequently discussed with the rest of the research team. Use of humour was identified as an indicator of youth-targeted messaging since youth perceived health advertisements with humour as positive and convincing [27, 28]. Although humour in public health messages is also an effective tool among the general population [29, 30], a meta-analysis reported that a younger population responded better to it and it has a more robust effect in messaging in younger people [31].

### Post type

Each post was assessed on whether it targeted youth and young adults using explicit or implicit messaging techniques. Posts were categorized as explicit if they fulfilled criteria 1 (specific mention of the targeted age group) *regardless* of meeting criteria 2 to 5. Posts were categorized as implicit if they met *any* of criteria 2 to 5 (i.e. (2) featuring actors or characters that look they may be in this age range; (3) mention of themes relevant to this age group; (4) use of humour; and (5) offering a fun or innovative way to practice PD that may appeal to youth.), but did not specifically mention the terms “youth,” “teen,” or “young adult,” “secondary students,” “post-secondary students,” or an age or age range within 16–29 years of age.

### Data extraction

Social media posts from each platform were scanned and collected by two independent reviewers between June and July 2020. The post type, post selection criteria, PHE information, link to the post, post caption, language of delivery, date posted, date accessed, post metrics and interactions were extracted and summarized for all posts on each platform using a data extraction sheet. Dyads compared their data entries and if consensus could not be reached, a third person was consulted to resolve any discrepancies. The definitions and data extraction process were pilot tested in a separate paper (manuscript in preparation).

### Interaction and post metrics

Interactions were operationalized as the frequency of response-related activities to social media posts. While the key names vary per platform, the underlying concept of interaction remains common. In this study, we counted the number of interactions for each individual post using the following metrics: (1) Facebook - reaction, share and comment; (2) Twitter - favorite, retweet and reply; (3) Instagram - like/love and comment, and (4) YouTube - video views, thumbs up or down and comment. For this study, video views were only considered in the total interaction count for YouTube, as it is consistently available for all posts. Video views from posts with embedded videos on Facebook and Instagram were excluded in the total interaction count for that post. All interactions were recorded at the time of the extraction (between June–July 2020).

### Behavior change techniques (BCTs)

The Behavior Change Technique Taxonomy version 1 (BCTTv1) [32] was used to code each of the posts. The BCTTv1 is organized into 93 individual BCTs organized into 16 categories, and the taxonomy lays the foundation for a reliable and standardized approach in classifying the active ingredient within PD messaging components. All data extractors completed formal BCT training, and posts were coded in duplicate for the presence or absence of BCTs. Coding between pairs was compared, and disagreements were resolved by consensus or with the perspective of a third reviewer.

### Data analysis

For each social media post, post descriptors, poster interest metrics, and BCT features were entered into an Excel sheet. Frequency counts and proportions were calculated to describe the data set.

## Results

**Fig. 1:**
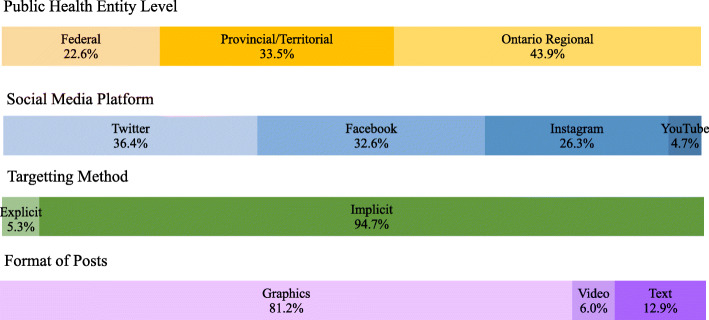
Characteristics of the posts with physical distancing messages for youth from social media platforms of Canadian public health entities (PHEs)

The study identified 319 posts with PD messaging targeted at youth and young adults from social media platforms of Canadian PHEs from April 1st until May 31st, 2020. The links and metrics of the social media accounts of PHEs that were included in this study are listed in Additional file 2. Figure 1 describes the characteristics of posts with PD messages targeting youth. Of the included posts, the highest proportion was from the Ontario regional public health units (43.9%), followed by provincial/territorial (33.5%) and federal (22.6%) PHEs. Out of all four platforms, Twitter (36.4%) and Facebook (32.6%) had the highest number of posts extracted and 81.2% of the posts included some form of graphics. With respect to post type, we found that the majority of posts were implicitly targeting youth, with only 5.3% of the posts explicitly mentioning youth.

Table 1 shows the breakdown of posts by their type, PHE level, and social media platform. The social media accounts from the federal PHEs had the highest proportion of explicitly targeted posts (8.3%), whereas the provincial/territorial PHEs had the lowest proportion (3.7%). The proportion of explicitly targeted posts ranged from 1.2 to 13.3% across different social media platforms. Although YouTube had the lowest number of extracted posts (4.7%), it had the highest number of posts across all platforms that explicitly targeted youth (13.3%). Twitter was the social media platform with the highest number of posts with PD messaging targeted at youth; however, only 6.9% of the posts targeted youth explicitly. Additional file 3 describes individual PD posts by each PHE.

**Table 1:** Social media posts by post type with PD messaging for youth aged 16–29 years

	Post Type	
	Explicit *n* = 17 (5.3%) n (%)	Implicit *n* = 302 (94.7%) n (%)
Public Health Entities		
Federal (*n* = 72)	6 (8.3)	66 (91.7)
Provincial/Territorial (*n* = 107)	4 (3.7)	103 (96.2)
Ontario Regional (*n* = 140)	7 (5.0)	133 (95.0)
Social Media Platform		
Twitter (*n* = 116)	8 (6.9)	108 (93.1)
Facebook (*n* = 104)	6 (5.8)	98 (94.2)
Instagram (*n* = 84)	1 (1.2)	83 (98.8)
YouTube (*n* = 15)	2 (13.3)	13 (86.7)

For each social media platform by PHE level and post type, Table 2 reports the interaction metrics for the single most interactive post in each category. As can be seen, the most interactive implicitly targeted posts consistently resulted in higher numbers of interactions compared to the most interactive explicitly targeted posts, regardless of social media platform. The explicitly targeted post with the highest number of interactions was a YouTube video from Northwest Territories Health and Social Services explaining PD; the video had accumulated 913 interactions, including video views (a screenshot of the post is available in Additional file 4). Excluding this YouTube video, two explicitly targeted posts from the federal PHEs had the highest number of interactions compared to other PHE levels, and both posts encouraged young Canadians to practice PD to do their part in flattening the curve on COVID-19 (Additional file 4).

**Table 2:** Interaction metrics for the single most interactive post by platform, PHE level, and post type

	Post Type					
	Explicit			Implicit		
Social Media Platform	Federal	Provincial/Territorial	Ontario Regional	Federal	Provincial/Territorial	Ontario Regional
Twitter	200	28	118	900	268	540
Facebook	338	12	124	1016	4155	510
Instagram	–	–	1	875	16	28,253
YouTube	–	913	–	–	2667	533

The implicitly targeted post with the highest number of interactions across the post types, PHE levels, and social media platforms was from the Toronto Public Health Instagram page (28,235 views: Table 2). In a short video posted on May 4th, the recommended 2 m or 6 ft distance for PD was described using lightsabers between 2 people in celebration of “May the Fourth (be with you),” a commemorative day of the Star Wars movie franchise (Additional file 4). The Facebook post with the highest number of interactions was from the Ministère de la Santé et des Services Sociaux (4155 views, Table 2). It was a 15-s video of a woman sitting on a bench encouraging viewers to practice PD while emphasizing the reasons to do so (i.e. protect yourself, your family, your community) with 369,000 views (Additional file 4).

Table 3 describes the proportion of posts with at least one BCT present. Out of 319 PD posts, three quarters (*n* = 237) contained one or more BCTs. Over 75% of the implicit posts (*n* = 228/302) contained one or more BCTs as compared to 52.9% of the explicit posts (*n* = 9/17). The highest proportion of explicitly targeted posts with one or more BCTs were extracted from the Ontario Regional public health units (85.7%) and were posted on Twitter (50.0%). The highest proportion of implicitly targeted posts were extracted from the federal PHEs (84.8%) and were posted on YouTube (92.3%). Additional file 5 elaborates Table 3 by each PHE.

**Table 3:** Posts with one or more BCT by post type, PHE level and social media platform

	Explicit (*n* = 17)		Implicit (*n* = 302)	
	Posts with BCT (*n* = 9; 52.9%) n (%)	Posts without BCT (*n* = 8; 47.1%) n (%)	Posts with BCT (*n* = 228; 75.5%) n (%)	Posts without BCT (*n* = 74; 24.5%) n (%)
Public Health Entity level				
Federal (*n* = 72)	0 (0.0)	6 (100.0)	56 (84.8)	10 (15.2)
Provincial/Territorial (*n* = 107)	3 (75.0)	1 (25.0)	84 (81.6)	19 (18.4)
Ontario Regional (*n* = 140)	6 (85.7)	1 (14.3)	88 (66.2)	45 (33.8)
Social Media Platform				
Twitter (*n* = 116)	4 (50.0)	4 (50.0)	85 (78.7)	23 (21.3)
Facebook (*n* = 104)	2 (33.3)	4 (66.7)	66 (67.3)	32 (32.7)
Instagram (*n* = 84)	1 (100.0)	0 (0.0)	65 (78.3)	18 (21.7)
YouTube (*n* = 15)	2 (100.0)	0 (0.0)	12 (92.3)	1 (7.7)

The specific types and frequency of BCTs are reported in Table 4. Sixteen individual BCTs representing 7 unique technique types and 551 BCTs representing 18 unique technique types were recorded in the explicit and implicit posts, respectively. Additional file 6 provides the definition and examples of BCTs in the context of youth-targeted PD messages in social media posts. *Instruction on how to perform the behavior* was the most frequent type of technique among both post types (explicit = 25.0% and implicit = 22.5%). Examples of this type of messaging included visual representations of figures standing 2 m apart. Another common type of technique was *restructuring the social environment* (explicit = 18.5% and implicit = 13.4%). This technique was defined as changing the social environment in order to perform PD and was seen in posts with recommendations of virtual get-togethers, such as Netflix parties, video calls, Zoom or phone calls, to limit face-to-face contact with others. Other BCTs that frequently appeared in explicit and implicit posts were *demonstration of the behavior* (providing an observable sample of the PD via graphics or videos), *information about health consequences* (providing information on health consequences of performing PD), and *distraction* (suggesting an alternate focus on attention to avoid situations where it is challenging to practice PD).

**Table 4:** Frequency of BCTs by post type

Specific Types of Behavioral Change Techniques	Post type	
	Explicit n (%)	Implicit n (%)
Goals and Planning		
Goal setting (behaviour)	0	5 (0.9)
Action planning	0	107 (18.8)
Social support		
Social support (unspecified)	0	4 (0.7)
Social support (emotional)	1 (6.3)	7 (1.4)
Shaping Knowledge		
Instruction on how to perform the behavior	4 (25.0)	124 (22.5)
Natural Consequences		
Information about health consequences	3 (18.8)	36 (6.5)
Information about social and environmental consequences	0	12 (2.2)
Comparison of Behaviour		
Demonstration of the behaviour	2 (12.5)	100 (18.1)
Associations		
Prompts	0	2 (0.4)
Repetition and substitution		
Behavior substitution	2 (12.5)	21 (3.8)
Comparison of Outcomes		
Credible sources	0	10 (1.8)
Reward and Threat		
Future Punishment	0	5 (0.9)
Regulation		
Reduce negative emotions	0	3 (0.5)
Antecedents		
Restructuring the physical environment	0	8 (1.5)
Restructuring the social environment	3 (18.8)	74 (13.4)
Avoidance/reducing exposure to cues for the behavior	0	15 (2.6)
Distraction	1 (6.3)	17 (3.1)
Identity		
Framing	0	1 (0.2)
Total Frequency of BCTs	16	551
Total Frequency of Unique Types of BCTs.	7	18

## Discussion

The current study aimed to analyze PD messaging targeted at youth aged 16–29 across different social media accounts belonging to various Canadian PHEs, particularly with the goal of assessing what content and format of social media post were engaged with the most. Additionally, these youth-targeted posts were examined for the presence of informed characteristics of behavior change interventions, and which appeared the most frequently. The main findings from this study highlight the lack of explicitly youth-targeted PD posts, the importance of choosing the appropriate social media platform and the absence of effective behaviour change messaging within these posts.

In July 2020, people aged 39 and below accounted for the surge in the number of daily COVID-19 cases across Canada [33]. The Chief Public Health Officer of Canada delivered a message shortly after asking young Canadians to continue following pandemic safety measures, especially PD. Although this message may have been heard by many, the results from this study revealed the lack of social media posts across all PHEs in which youth and young adults are explicitly mentioned. Previous online health promotion campaigns have shown that using a targeted method on social media has the capacity to reach a much larger population and subgroup (i.e. youth) as compared to generalized campaigns [34, 35]. Very few posts in this study addressed situations that some youth may be facing, such as housing instability, homelessness, domestic violence, and substance abuse, that may be even more difficult during the pandemic. By increasing the diversity of topics in social media PD campaigns, it can help prevent accidental COVID-19 spread due to the lack of knowledge on relevant topics [36, 37]. Social media has also been shown to be a better platform in reaching specific subgroups as it allows users to interact with one another, create online social networks and influence behaviour. A recent study found that the use of hashtags (#) in social media campaigns and posts enhanced visibility and reach among teens [38]. Many of the PHE accounts used the same post multiple times on different days. This may be seen as either an advantage or a disadvantage; by repeating the same post, the public can be reminded of the importance of PD however, if the post is not being engaged with or is not effective, PHEs miss the opportunity to improve their messaging and convey their message. By using this powerful tool to their benefit, PHEs can allow youth and young adults to influence one another about the perceived behavior being spoken about (in this case, PD) across platforms as the norm they and their age group should be following [13].

Social media use is incredibly common, with 96% of Canadian youth aged 16–24 connecting to these platforms on a daily basis [39]. Facebook remains the most widely used social media platform in Canada; however, users aged 18–24 dropped 11% from 2017 to 2020 (from 95% in 2017 to 84% in 2020) [40]. This seems to be a sign that this age group is moving towards other platforms. Although Twitter only has about half its users visit the platform every day, young people aged 18–24 (65%) and 25–34 (54%) are its most prevalent users [40]. However, there was only a 5% increase in the number of new users under 25 years; this suggests that the platform is becoming less relevant for this age group [40]. The highest number of extracted posts were from Facebook (32.6%) and Twitter (36.4%). This is an important result for PHEs that may want to target a younger age group but may not be selecting the correct platform. Instagram and YouTube have over 65% of their total users visiting daily. Furthermore, 18–24 year-olds are the dominant group on Instagram and make up 89% of its audience [40]. Over time, Instagram has become increasingly more popular in this age group, having the highest growth rate in new users aged 18–24 among all platforms between 2017 and 2020 (from 67% in 2017 to 89% in 2020) [40]. Only one explicit post was extracted from this platform in this study. Given the high number of youth on Instagram, PHEs may want to consider targeting youth on this site. YouTube is the third most popular social media platform in Canada and is dominated by users 13 and over [41]. In a 2020 report, it was reported that regardless of age, 60% of youth spent their spare time watching video content on YouTube [42]. In total, they spend 1–3 h/day on an average day using this social media platform [43]. Given the high concentration of youth on YouTube and the amount of time spent on this platform, PHEs may want to consider using YouTube in future health promotion campaigns in order to target the audiences 18 and younger.

Including and using BCTs in social media posts for health interventions is an important tool that should be used in order to design more effective and successful interventions and specify and report intervention content in a consistent manner [19, 20]. Previous studies have shown that the use of BCTs in social media or online interventions can often elicit the desired behavior change in youth and young adults [44–47]. Furthermore, posts which directly mention the target population may need to use less BCTs compared to implicit or generalised posts to engage the same behavior [48, 49]. An implicit post may need to use both *demonstration of the behavior* and *instruction on how to perform the behavior* BCTs to target youth in their post as well as inform on the rules and regulations of PD.

*Instruction on how to perform the behavior* was the BCT that appeared most often among both explicit and implicit posts. Observing PD was likely the most important message for PHEs to disseminate to the public at the beginning of the pandemic; they needed to advise Canadians on what this new term meant and how to execute it correctly, as this public health strategy has been key in controlling the pandemic [50, 51] This can also apply to the *demonstration of the behavior* BCT, as many simple and useful graphics were created by public health authorities in order to rapidly inform the population about PD. These two BCTs are often the most commonly used as they are educational and provide the base instruction to the target audience [52]. Furthermore, they can be easily and implicitly be incorporated into messaging, especially among interventions that require instruction of a new behavior [52]. Previous research using BCT messaging among adults found that those that provided instruction were key techniques in eliciting behaviour change compared to messages which did not contain these BCTs [53, 54].

The COVID-19 pandemic and PD have been particularly challenging for youth for whom social connections with peers and experiencing milestones, such as prom and graduation, are especially important [55–58]. PD-related posts extracted from social media accounts of PHEs at all jurisdiction levels seem to address this by using both the *restructuring the social environment* and the *social support* BCTs. These appeared quite frequently and aligned with the common difficulties that arose during the pandemic among youth and young adults who no longer were able to engage in social interactions in the same way and needed to lean on one another differently. Another common BCT used in both post types was *information about health consequences*, which allowed PHEs to inform youth about the consequences of this novel disease as a way to motivate behaviour change with respect to practicing PD [59]. Use of this BCT could be an effective way to reach youth who may have disregarded their vulnerability to COVID-19 throughout the pandemic due to a reported lower risk of infection for their age group [59, 60]. Although BCT components were identified in each extracted post, measuring their offline impact and effectiveness in promoting PD behaviour in our target audience (i.e., the number of individuals performing PD) is challenging. A systematic review by Maher et al. [61] found social media to be the main method for PHEs to deliver health behavior change interventions; however, the study could not determine whether or not this translated to behavior change due to small effect sizes of the assessed studies [61]. Future studies should examine whether interactions on social media translate into real behavioral change. Additionally, there is mixed evidence whether BCTs alone are effective at promoting behavioural change, or whether other interventions plus BCTs are more effective [62]. As this area of research is still limited, future research should examine these relationships.

### Strengths and limitations

This is the first study to our knowledge that has examined youth-targeted PD messages on social media and the use of BCT in these messages. The content analysis, including post extraction and BCT analysis, was conducted by two reviewers. Each reviewer was trained in the BCT framework and consulted with several experts in the field of behavior change and implementation science. Using the official BCT coding framework, a consensus for the types of posts and which BCT they should fall under was achieved a priori to BCT extraction. BCT coding was adapted to social media posts that addressed PD, which has not been previously done before in the context of the COVID-19 pandemic. Furthermore, consumers of social media within the age range of interest were consulted in order to develop the criteria for implicit and explicit youth-targeted posts. Another strength includes the representation of the pandemic during the study period: a large sample of posts were extracted with a wide range of post content and format over a significant period when these PD measures were first being introduced.

This study has certain limitations. Although a significant number of posts were captured across different jurisdiction levels and social media platforms, due to the cross-sectional nature of this study, the findings are limited to the predetermined time period of extraction and are not generalizable to other time frames or PHEs. During a global pandemic, the information available about PD changes daily; therefore, choosing a different time frame could have resulted in a different number of total posts extracted as well as the number of BCTs present. Future research should examine the evolution of PD messages (type of post, number of interactions) on social media from public health campaigns throughout the pandemic. There is a possibility that some social media posts were removed or deleted by the PHEs by the time of data extraction and would have been missed. Another limitation was the over-representation of the province of Ontario in our sample. Only the regional public health units within the province of Ontario were examined, and thus the majority of extracted posts originated from Ontario.

The number of reactions and interactions from each post could have been influenced by the length of time it had been live; for instance, social media posts at the beginning of April 2020 could have had more interactions than those at the end of May 2020. However, research conducted on engagement periods for marketing strategies on social media have found that a majority of interactions occur within the first 1–3 h [63, 64]. As all the posts from the PHEs were online for 2–3 months at the time of extraction, this should have minimally impacted the interaction counts. The number of users on the social media platform could have also influenced the number of interactions. Facebook is the most used social media platform with over 2.25 billion users, and the posts themselves can be text, graphics and/or videos [65]. Thus, users have the opportunity to interact with more types of posts than just graphics and videos such as on Instagram and YouTube or primarily text on Twitter. Due to limited access to social media analytics, it was not possible to assess which age groups interacted with extracted posts and thereby determine whether the target audience engaged more with social media posts that implicitly or explicitly targeted youth. Generally, youth and young adults are more likely to use social media as compared to older adults; however, some platforms are much more popular among youth [66].

The BCT analysis was applied retrospectively to published public posts. It is unclear if inclusion of BCT into public health messaging was purposeful or not. However, given the evidence of effectiveness of incorporating BCTs and other advancements from the implementation science discipline in other contexts, PHEs are encouraged to systematically explore their use of these scientific tools [21]. Collaborations such as the OPH-SEPH collaborative working group may provide an exemplar of how cross-sectoral collaboration may enable learnings for all parties and greater impact than if the PHEs work alone (manuscript in preparation). Future studies should also examine the collaboration of the PHEs within and across levels (i.e., federal, provincial and regional); greater collaboration between PHEs for their communication plans during an emergency situation may lead to greater consistency in messages and great impact.

## Conclusion

PHEs that are planning to launch social media campaigns related to COVID-19 and PD should consider who their target population is and then examine which social media platform may be best to reach these users. Furthermore, choosing a platform that accommodates multiple types of posts, such as graphics, videos and text (Instagram), as compared to a 1-media type (YouTube) social media platform, may allow for more engagement with the audience.

Using behavior change domains within social media campaigns posts should be considered while designing posts to deliver health interventions/messaging. Creating posts that not only appeal to youth but also explicitly address them may be a way to increase engagement. Posts with engaging graphics and clear BCTs may increase the likelihood that the target audience sees it and engages with it.

## Supplementary Information

**Additional file 1.** Overview of Canadian PHEs. The overview of Canadian PHE levels and the number of social media accounts owned by each level.

**Additional file 2.** Social Media Metrics of Canadian PHEs. The metrics (numbers of subscribers and followers) and URLs of social media accounts owned by the Canadian PHEs.

**Additional file 3.** Number of social media posts by post type with PD messaging for youth aged 16–29 years for each PHE. Further elaborates Table 1 and shows the frequency of social media posts with PD messaging for youth by individual PHE and post type.

**Additional file 4.** Examples of social media posts with PD messages for youth. Screenshots of the most interactive and publicly available social media posts with PD messages for youth (4 total).

**Additional file 5.** Number of posts with one or more BCT as a function of the post type for each PHE. Further elaborates Table 3 and shows the frequency of social media posts with one or more BCT by individual PHE and post type.

**Additional file 6.** Definitions and examples of BCTs found in social media posts with youth-targeted PD messages. Provides definitions of BCTs that were identified from the social media posts and examples in context of PD messages.

## Data Availability

The datasets used and/or analysed during the current study are available from the corresponding author on reasonable request.

## Abbreviations

COVID-19: Coronavirus disease 2019
PD: Physical distancing
BCT: Behavioral change techniques
PHE: Public health entities
