# Supplementary figures and images for: Physical distancing messages targeting youth on the social media accounts of Canadian public health entities and the use of behavioral change techniques
Source: BMC Public Health. 2021 Sep 7;21:1634. doi: 10.1186/s12889-021-11659-y (PMC8422061; doi:10.1186/s12889-021-11659-y)

**ADDITIONAL FILE 1:** Overview of Canadian PHEs.

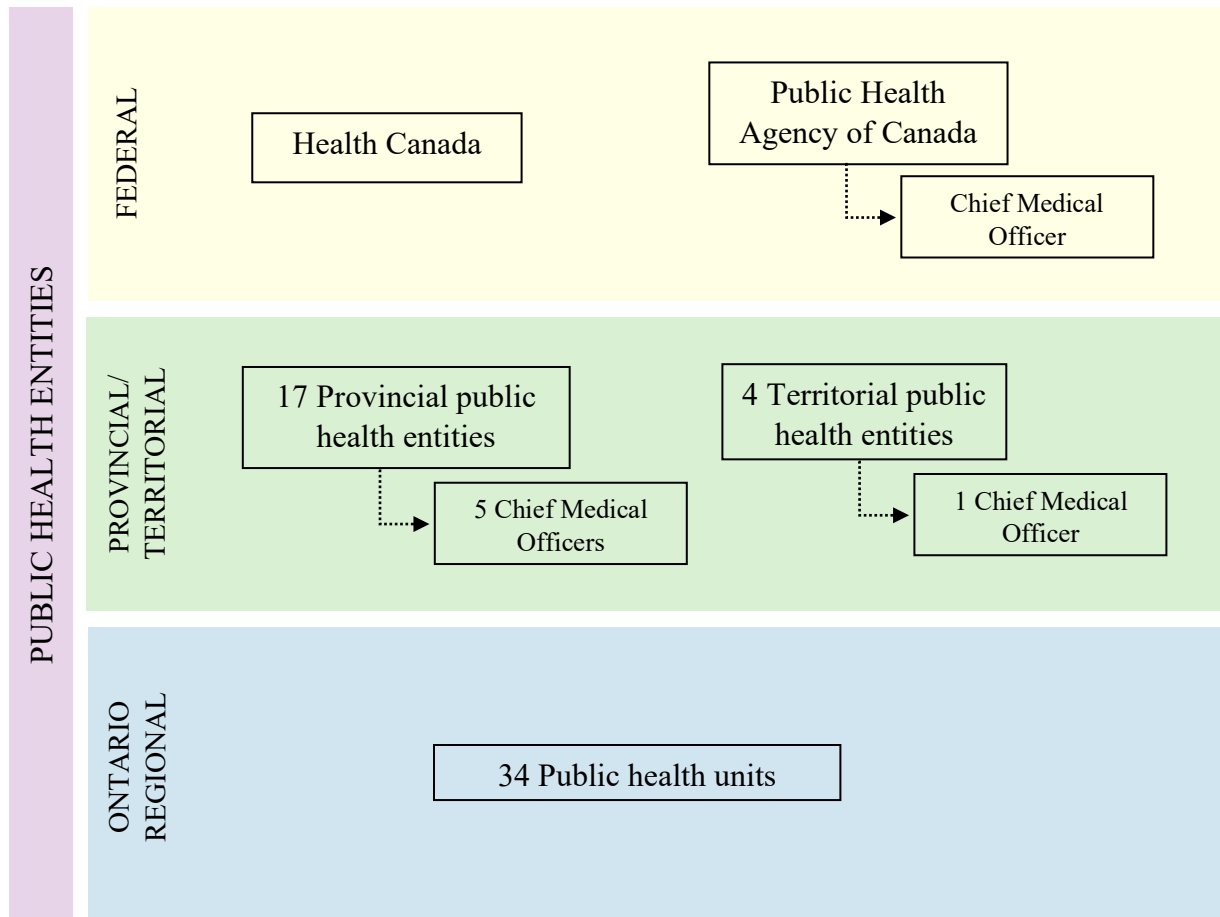

Supplement: Supplementary file 1 — Additional file 1. Overview of Canadian PHEs. The overview of Canadian PHE levels and the number of social media accounts owned by each level. [file 12889_2021_11659_MOESM1_ESM.pdf]
