# Supplementary material for: Physical distancing messages targeting youth on the social media accounts of Canadian public health entities and the use of behavioral change techniques
Source: BMC Public Health. 2021 Sep 7;21:1634. doi: 10.1186/s12889-021-11659-y (PMC8422061; doi:10.1186/s12889-021-11659-y)
Supplement: Supplementary file 2 — Additional file 2. Social Media Metrics of Canadian PHEs. The metrics (numbers of subscribers and followers) and URLs of social media accounts owned by the Canadian PHEs. [file 12889_2021_11659_MOESM2_ESM.pdf]

ADDITIONAL FILE 2: Social Media Metrics of Canadian PHEs.

Health Unit Name	Links				Numbers of subscribers and followers[i]				
	Facebook page URL	Twitter handle/URL	Instagram handle/URL	YouTube channel URL	Facebook Likes	Facebook Followers	Twitter Followers	Instagram Followers	YouTube Subscribers
**Ontario Regional**									
Algoma Public Health Unit	https://www.facebook.com/algomahealth/	https://twitter.com/AlgomaHealth	-	https://www.youtube.com/user/AlgomaPublicHealth	8,756	8,901	1,803	-	606
Brant County Health Unit	https://www.facebook.com/branthealthunit/	https://twitter.com/BrantHealthUnit	https://www.instagram.com/branthealthunit/	https://www.youtube.com/user/branthealthunit	2,437	2,700	2,492	1,070	25
Chatham-Kent Health Unit	https://www.facebook.com/CKPublicHealth/	https://twitter.com/ckpublichealth	https://www.instagram.com/ckpublichealth/	https://www.youtube.com/user/ckphu	4,211	4,592	2,065	1,419	655
Durham Region Health Department	https://www.facebook.com/durhamregionhealth/	https://twitter.com/DurhamHealth	https://www.instagram.com/durhamregionhealth/	https://www.youtube.com/user/RegionofDurham	9,626	9,798	23,000	2,924	2,360
Eastern Ontario Health Unit	https://www.facebook.com/EOHUhealth/	https://twitter.com/EOHU_tweet	https://www.instagram.com/eohucomm	https://www.youtube.com/user/EasternOntarioHealth	5,016	5,644	1,717	1,018	6,590
Grey Bruce Health Unit	https://www.facebook.com/greybrucepublichealth/	https://twitter.com/GBPublicHealth	https://www.instagram.com/gbpublichealth/	https://www.youtube.com/user/GreyBruceHealthUnit	4,803	5,326	2,674	1,235	2,870
Haldimand-Norfolk Health Unit	https://www.facebook.com/hnhealthunit/	https://twitter.com/HNHealthUnit	https://www.instagram.com/hnhealthunit/	https://www.youtube.com/user/hnhucommunications	3,204	3,563	1,057	310	56
Haliburton, Kawartha, Pine Ridge District Health Unit	https://www.facebook.com/HKPRDHU/	https://twitter.com/hkprdhu	-	https://www.youtube.com/user/HKPRDHU	1,534	1,807	1,713	-	614
Halton Region Health Department	https://www.facebook.com/RegionofHalton/	https://twitter.com/regionofhalton	https://www.instagram.com/regionofhalton/?hl=en	https://www.youtube.com/user/RegionOfHalton	5,954	6,644	16,000	-	425

Hamilton Public Health Services[ii]	https://www.facebook.com/pages/Hamilton-Public-Health-Svc/305759659769249	https://twitter.com/cityofhamilton	https://www.instagram.com/cityofhamilton/	https://www.youtube.com/user/InsideCityofHamilton	-	-	78,800	24,200	3,760
Hastings and Prince Edward Counties Health Unit	https://www.facebook.com/hpepublichealth/	https://twitter.com/HPEPublicHealth	-	https://www.youtube.com/channel/UCikA8QusuOGTQ-WJSuDWCiw	7,708	8,066	1,549	-	36
Huron Perth County Health Unit	https://www.facebook.com/HuronPerthPublicHealth/	https://twitter.com/hppublichealth	https://www.instagram.com/thinkhuronperth/	https://www.youtube.com/channel/UCHiZbV6cQQ2WhUas-TXyXKg	4,380	5,073	1,907	184	34
Kingston, Frontenac and Lennox & Addington Health Unit	https://www.facebook.com/kflapublichealth/	https://twitter.com/kflaph	https://www.instagram.com/kflaph/	https://www.youtube.com/user/KFLAPublicHealth	6,075	6,785	6,238	1,621	155
Lambton Health Unit	https://www.facebook.com/lambtonpublichealth/	https://twitter.com/lambton_ph	-	https://www.youtube.com/channel/UCMFFyWPd6Ii4-arU3iviYHA	4,165	4,410	735	-	Not listed
Leeds, Grenville and Lanark District Health Unit	https://www.facebook.com/LGLHealthUnit/	https://twitter.com/LGLHealthUnit	-	https://www.youtube.com/user/LGLHealthUnit	4,679	5,107	886	-	43
Middlesex-London Health Unit	https://www.facebook.com/middlesex.london.health.unit/	https://twitter.com/MLHealthUnit	https://www.instagram.com/mlhealthunit/	https://www.youtube.com/user/mlhealthunit	12,064	12,780	14,100	5,428	1,820
Niagara Region Public Health Department[iii]	https://www.facebook.com/niagararegion/	https://twitter.com/NRPublicHealth	https://www.instagram.com/niagarafallsontario/	https://www.youtube.com/user/NiagaraRegionCA	22,340	24,113	22,700	4,152	Not listed
North Bay Parry Sound District Health Unit	https://www.facebook.com/NorthBayParrySoundDistrictHealthUnit/	https://twitter.com/nbpsdhealthunit	-	https://www.youtube.com/user/NBPSHealthUnit	4,028	4,434	1,401	-	50
Northwestern Health Unit	https://www.facebook.com/TheNWHU/	https://twitter.com/TheNWHU	https://www.instagram.com/thenwhu/	https://www.youtube.com/user/TheNWHU	5,134	5,488	808	606	46
Ottawa Public Health	https://www.facebook.com/ottawahealth/	https://twitter.com/ottawahealth	https://www.instagram.com/ottawahealthsante/	https://www.youtube.com/user/ottawahealthsante	35,098	38,085	79,600	24,600	1,780

Peel Public Health[iv]	https://www.facebook.com/regionofpeel/	https://twitter.com/regionofpeel	https://www.instagram.com/peelregion.ca/?hl=en	https://www.youtube.com/user/theregionofpeel	3,214	3,586	31,000	2,345	3,390
Peterborough Public Health	https://www.facebook.com/Ptbohealth/	https://twitter.com/Ptbohealth	https://www.instagram.com/ptbohealth/	https://www.youtube.com/user/PCCHUHealthUnit	2,493	2,866	3,393	1,368	23
Porcupine Health Unit[v]	https://www.facebook.com/yourPHU/	https://twitter.com/porcupinehu?lang=en	-	https://www.youtube.com/user/PorcupineHealth	5,528	6,070	448	-	52
Public Health Sudbury & Districts	https://www.facebook.com/PublicHealthSD/	https://twitter.com/ROWPublicHealth	https://www.instagram.com/rowpublichealth/?hl=en	https://www.youtube.com/user/regionofwaterloo	7,899	8,555	3,377	-	179
Region of Waterloo, Public Health[vi]	https://www.facebook.com/RegionWaterloo/	https://twitter.com/RCDHealthUnit	-	https://www.youtube.com/channel/UCWQ1CpM4IbJNNnNI8MLd-aA/featured	3,451	3,810	6,860	1,650	661
Renfrew County and District Health Unit	https://www.facebook.com/RCDHealthUnit/	https://twitter.com/SMDhealthunit	https://www.instagram.com/simcoemuskokahealth/	https://www.youtube.com/user/smdhealthunit/featured	3,023	3,279	622	-	76
Simcoe Muskoka District Health Unit	https://www.facebook.com/Simcoe.Muskoka/	https://twitter.com/SW_PublicHealth	https://www.instagram.com/swpublichealth/	https://www.youtube.com/user/ESTPH1	8,818	10,043	3,347	348	279
Southwestern Public Health	https://www.facebook.com/swpublichealth/	https://twitter.com/PublicHealthSD	-	https://www.youtube.com/PublicHealthSD	8,129	8,637	1,976	712	22
Thunder Bay District Health Unit	https://www.facebook.com/TBDHealthUnit/	https://twitter.com/TBDHealthUnit	https://www.instagram.com/tbdhealthunit/	https://www.youtube.com/user/TBDHealthUnit	11,790	13,514	3,181	3,198	138
Timiskaming Health Unit	https://www.facebook.com/Timiskaming-Health-Unit-108069779257419/	https://twitter.com/TimiskamingHU	-	https://www.youtube.com/channel/UCpwZynPfKEBoT58z4FDu_nA	3,189	3,739	176	-	7
Toronto Public Health	https://www.facebook.com/torontopublichealth/	https://twitter.com/TOPublicHealth	https://www.instagram.com/cityofto/	https://www.youtube.com/user/thecityoftoronto	28,070	31,779	400,400	116,000	10,600

Wellington-Dufferin-Guelph Health Unit	https://www.facebook.com/WDGPublicHealth/	https://twitter.com/WDGPublicHealth	https://www.instagram.com/wdgpublichealth/	https://www.youtube.com/c/WDGPublicHealth	3,316	3,751	5,532	1,506	76
Windsor-Essex County Health Unit	https://www.facebook.com/TheWECHU/	https://twitter.com/TheWECHU	https://www.instagram.com/thewechu/?hl=en	https://www.youtube.com/user/wechealthunit	14,901	18,732	3,907	615	Not listed
York Region Public Health Services[vii]	https://www.facebook.com/YorkRegionGovt/	https://twitter.com/YorkRegionGovt	https://www.instagram.com/YorkRegionGovt/	https://www.youtube.com/YorkRegionGovt	21,597	23,930	22,500	9,535	1,450
Provincial/Territorial									
Alberta Ministry of Health (Alberta Health)	-	https://twitter.com/goahealth	-	https://www.youtube.com/channel/UCnuHHh1V4fubDUY_8YN8ysA	-	-	18,417	-	27,000
Alberta Health Services	https://www.facebook.com/albertahealthservices/	https://twitter.com/AHS_media?ref_src=twsrc%5Egoogle%7Ctwcamp%5Eserp%7Ctwgr%5Eauthor	https://www.instagram.com/albertahealthservices/	https://www.youtube.com/user/AHSChannel	59,590	63,800	34,006	28007	8900
Deena Hinshaw - The chief medical officer of Alberta		https://twitter.com/CMOH_Alberta			-	-	87,783	-	-
British Columbia Ministry of Health	-	-	-	https://www.youtube.com/user/ProvinceofBC	-	-	-	-	10000
Provincial Health Services Authority	https://www.facebook.com/ProvincialHealthServices/	https://twitter.com/PHSAofBC	https://www.instagram.com/phsa.bc/	https://www.youtube.com/user/ProvHealthServAuth	5,900	6,527	12,172	3767	Not listed
BC Centre for Disease Control	-	https://twitter.com/cdcofbc	-	https://www.youtube.com/user/thebccdc/featured	-	-	28,223	-	336
Manitoba Health, Seniors and Active Living[viii]	https://www.facebook.com/ManitobaGovernment/	https://twitter.com/mbgov	-	https://www.youtube.com/user/ManitobaGovernment	28,464	30,544	33,037	-	4480
Brent Roussin - The chief medical officer of Manitoba		https://twitter.com/roussin_brent					5,512	-	-

New Brunswick Department of Health	-	https://twitter.com/NBHealth	-	https://www.youtube.com/playlist?list=PLA8D47F3CEFB8D4B6	-	-	4,687	-	15,400
Newfoundland and Labrador - Department of Health and community services	-	https://twitter.com/hcs_govnl?lang=en	-	https://www.youtube.com/channel/UCcXvPIKQKhu0YenR4YgOD2Q	-	-	6,994	-	2070
Janice Fitzgerald - The chief medical officer of Newfoundland and Labrador		https://twitter.com/CMOH_NL			-	-	11,115	-	-
Kami Kandola - The chief medical officer of NWT		https://twitter.com/NWT_CPHO			-	-	1,594		-
Northwest Territories Health and Social Services Authority	https://www.facebook.com/NTHSSA/?pageid=374322463359625&ftentidentifier=649223842536151&padding=0	https://twitter.com/nthssa	-	https://www.youtube.com/user/HSSCommunications	712	756	10	-	Not listed
Nova Scotia Department of Health and Wellness	https://www.facebook.com/NovaScotiaHealthAndWellness	https://twitter.com/nshealth	-	https://www.youtube.com/user/NSDHW	15,799	17,073	15,269	-	Not listed
Robert Strang - The chief medical officer of Nova Scotia		https://twitter.com/StrangRobert			-	-	8,004	-	-
Nova Scotia Health Authority	https://www.facebook.com/NovaScotiaHealthAuthority	https://twitter.com/healthns	https://www.instagram.com/novascotiahealthauthority/	https://www.youtube.com/channel/UCLd2y0L2paPZLTjk0LnUQlQ	19,264	20,613	7,088	3,643	129
Nunavut Department of Health and Social Services[ix]	https://www.facebook.com/GovofNunavut/	https://twitter.com/GovofNunavut	-	https://www.youtube.com/user/GovernmentofNunavut	12,872	13,249	7,099	-	159
Public Health Ontario	https://www.facebook.com/PublicHealthON	PublicHealthON	-	https://www.youtube.com/channel/UCVHo7YRHEGDvc9JtqYA16UQ	5,967	6,469	43,156	NA	0
Prince Edward Island Department of Health and Wellness[x]	https://www.facebook.com/govpe	https://twitter.com/PEIwellness	-	https://www.youtube.com/user/govpeca/featured	28,809	41,624	5165	-	4880

Health PEI		https://twitter.com/Health_PEI?ref_src=twsrc%5Egoogle%7Ctwcamp%5Eserp%7Ctwgr%5Eauthor	-	-	-	-	4,976	-	-
Institut national de santé publique du Québec	https://www.facebook.com/inspq	https://twitter.com/INSPQ	https://www.instagram.com/inspquebec/?hl=en	https://www.youtube.com/user/INSPQuebec	13,522	14,948	9651	631	425
Quebec Ministry of Health and Social Services | Ministère de la Santé et des Services sociaux	https://www.facebook.com/SanteServicesSociauxQuebec/	https://twitter.com/sante_qc?lang=en"	-	https://www.youtube.com/channel/UCixMqPbpL6dCQqHxDbgoUfQ	38,629	42,253	39,723	-	2460
Horacio Arruda - The chief medical officer of Quebec		https://twitter.com/ArrudaHoracio			-	-	27,275	-	-
Saskatchewan Ministry of Health (Saskatchewan Health)			-	https://www.youtube.com/channel/UC9Wg6ZI0aM3Mx2KrWQApTwA/discussion?lc=Ugzzn7fpyDtpTyZDMCB4AaABAg	-	-	-	-	124
Saskatchewan Health Authority	https://www.facebook.com/SaskHealthAuthority/	https://twitter.com/saskhealth	-	https://www.youtube.com/channel/UC3KEimyU6nCmwYLpieHp8Fw	31,157	33,206	6,988	-	146
Yukon Department of Health and Social Services	https://www.facebook.com/yukonhss	https://twitter.com/hssyukon	https://www.instagram.com/yukonhss/?hl=en	https://www.youtube.com/c/hssyukongovernment	3,572	3,760	1,365	274	305
Federal									
Health Canada/PHAC	https://www.facebook.com/HealthyCdns	https://www.instagram.com/HealthyCdns/	https://twitter.com/GovCanHealth	https://www.youtube.com/user/HealthyCdns	163,181	337,937	322,530	39,974	6830
Health Canada/PHAC - French	https://www.facebook.com/CANenSante/	https://www.instagram.com/CANensante/	https://twitter.com/GouvCanSante	https://www.youtube.com/channel/UCYa9l5DZCq9-2ykCUiAicRg	62,450	154,503	28,606	1808	1610
Theresa Tam - The Chief Public Health Officer of Canada		https://twitter.com/CPHO_Canada			-	-	202,777	-	-

[i] All social media metrics were updated between May 28 and June 30
[ii] All social media accounts hosted by the city of Hamilton
[iii] All social media accounts hosted by the city of Niagara
[iv] All social media accounts hosted by the Region of Peel
[v] Instagram account private
[vi] All social media accounts hosted by the Region of Waterloo
[vii] All social media accounts hosted by the regional municipality of York
[viii] All social media accounts hosted by the Government of Manitoba
[ix] All social media accounts hosted by the Government of Nunavut
[x] The embedded Social media links were for Gov of PEI, not for PHE. It has separate twitter account for health which was not embedded
