# Supplementary material for: Physical distancing messages targeting youth on the social media accounts of Canadian public health entities and the use of behavioral change techniques
Source: BMC Public Health. 2021 Sep 7;21:1634. doi: 10.1186/s12889-021-11659-y (PMC8422061; doi:10.1186/s12889-021-11659-y)
Supplement: Supplementary file 3 — Additional file 3. Number of social media posts by post type with PD messaging for youth aged 16–29 years for each PHE. Further elaborates Table 1 and shows the frequency of social media posts with PD messaging for youth by individual PHE and post type. [file 12889_2021_11659_MOESM3_ESM.pdf]

ADDITIONAL FILE 3: Number of social media posts by post type with PD messaging for youth aged 16-29 years for each PHE.

		Post type	
		Explicit n=17 (5.3%)	Implicit n=302 (94.7%)
PUBLIC HEALTH ENTITIES			
Federal			
	Health Canada/PHAC	3	32
	Health Canada/PHAC - French	3	32
	Theresa Tam - The Chief Public Health Officer of Canada	0	2
Provincial/Territorial			
	Alberta Ministry of Health (Alberta Health)	0	5
	Alberta Health Services	0	9
	Provincial Health Services Authority	0	9
	BC Centre for Disease Control	1	4
	Manitoba Health, Seniors and Active Living [1]	0	5
	New Brunswick Department of Health	0	5
	Kami Kandola - The chief medical officer of NWT	0	9
	Northwest Territories Health and Social Services Authority	2	6
	Prince Edward Island Department of Health and Wellness [2]	0	15
	Institut national de santé publique du Québec	0	1
	Quebec Ministry of Health and Social Services | Ministère de la Santé et des Services sociaux	0	2
	Saskatchewan Health Authority	0	14
	Yukon Department of Health and Social Services	1	19
Ontario Regional			
	Algoma Public Health Unit	0	4
	Brant County Health Unit	0	4
	Chatham-Kent Health Unit	1	3
	Durham Region Health Department	0	2
	Eastern Ontario Health Unit	0	7
	Grey Bruce Health Unit	0	4
	Haliburton, Kawartha, Pine Ridge District Health Unit	0	5
	Hamilton Public Health Services [3]	0	1
	Hastings and Prince Edward Counties Health Unit	0	1
	Huron Perth County Health Unit	0	2

Kingston, Frontenac and Lennox & Addington Health Unit	0	1
Lambton Health Unit	0	3
Leeds, Grenville and Lanark District Health Unit	0	2
Middlesex-London Health Unit	0	7
Niagara Region Public Health Department [4]	0	7
North Bay Parry Sound District Health Unit	0	1
Northwestern Health Unit	0	12
Ottawa Public Health	4	23
Peel Public Health [5]	0	2
Peterborough Public Health	0	4
Public Health Sudbury & Districts	0	1
Simcoe Muskoka District Health Unit	0	2
Thunder Bay District Health Unit	1	13
Toronto Public Health	1	13
Wellington-Dufferin-Guelph Health Unit	0	4
York Region Public Health Services [6]	0	5

[1] All social media accounts hosted by the Government of Manitoba

[2] The embedded Social media links were for Gov of PEI, not for PHE. It has a separate twitter account for health which was not embedded

[3] All social media accounts hosted by the city of Hamilton

[4] All social media accounts hosted by the city of Niagara

[5] All social media accounts hosted by the Region of Peel

[6] All social media accounts hosted by the regional municipality of York

The table only includes the frequency of the PHU that has at least 1 post in any category. The following public health units did not have any social media posts with PD messaging and are not included in the table:

- Ontario regional: Haldimand-Norfolk Health Unit, Halton Region Health Department, Porcupine Health Unit, Region of Waterloo, Public Health, Renfrew County and District Health Unit, Southwestern Public Health, Windsor-Essex County Health Unit, Timiskaming Health Unit.

- Provincial/Territorial: Deena Hinshaw - The chief medical officer of Alberta, British Columbia Ministry of Health, Brent Roussin - The chief medical officer of Manitoba, Newfoundland and Labrador - Department of Health and community services, Janice Fitzgerald - The chief medical officer of Newfoundland and Labrador, Nova Scotia Department of Health and Wellness, Robert Strang - The chief medical officer of Nova Scotia, Nova Scotia Health Authority, Nunavut Department of Health and Social Services, Public Health Ontario, Health PEI, Horacio Arruda - The chief medical officer of Quebec, Saskatchewan Ministry of Health (Saskatchewan Health).
