# Supplementary material for: Physical distancing messages targeting youth on the social media accounts of Canadian public health entities and the use of behavioral change techniques
Source: BMC Public Health. 2021 Sep 7;21:1634. doi: 10.1186/s12889-021-11659-y (PMC8422061; doi:10.1186/s12889-021-11659-y)
Supplement: Supplementary file 4 — Additional file 4. Examples of social media posts with PD messages for youth. Screenshots of the most interactive and publicly available social media posts with PD messages for youth (4 total). [file 12889_2021_11659_MOESM4_ESM.pdf]

ADDITIONAL FILE 4: Examples of social media posts with PD messages for youth and young adults.

Youtube:
https://www.youtube.com/watch?v=c8BDGV5bABI

Twitter:
https://twitter.com/GovCanHealth/status/1253670667007995904

Instagram:
https://www.instagram.com/p/B_w-D8RFjWp/

Facebook:
https://www.facebook.com/watch/?v=246737823409773
