# Supplementary material for: Physical distancing messages targeting youth on the social media accounts of Canadian public health entities and the use of behavioral change techniques
Source: BMC Public Health. 2021 Sep 7;21:1634. doi: 10.1186/s12889-021-11659-y (PMC8422061; doi:10.1186/s12889-021-11659-y)
Supplement: Supplementary file 5 — Additional file 5. Number of posts with one or more BCT as a function of the post type for each PHE. Further elaborates Table 3 and shows the frequency of social media posts with one or more BCT by individual PHE and post type. [file 12889_2021_11659_MOESM5_ESM.pdf]

ADDITIONAL FILE 5: Number of posts with one or more BCT as a function of the post type for each PHE.

	Post type			
	Explicit (n=17)		Implicit (n=302)	
	Posts with BCT (n=9)	Posts without BCT (n=8)	Posts with BCT (n=228)	Posts without BCT (n=74)
PUBLIC HEALTH ENTITIES				
Federal				
Health Canada/PHAC	0	3	27	5
Health Canada/PHAC - French	0	3	27	5
Theresa Tam - The Chief Public Health Officer of Canada	0	0	2	0
Provincial/Territorial				
Alberta Ministry of Health (Alberta Health)	0	0	5	0
Alberta Health Services	0	0	6	3
Provincial Health Services Authority	0	0	7	2
BC Centre for Disease Control	1	0	3	1
Manitoba Health, Seniors and Active Living [1]	0	0	0	5
New Brunswick Department of Health	0	0	5	0
Kami Kandola - The chief medical officer of NWT	0	0	7	2
Northwest Territories Health and Social Services Authority	2	0	5	1
Prince Edward Island Department of Health and Wellness [2]	0	0	13	2
Institut national de santé publique du Québec	0	0	1	0
Quebec Ministry of Health and Social Services | Ministère de la Santé et des Services sociaux	0	0	2	0
Saskatchewan Health Authority	0	0	11	3
Yukon Department of Health and Social Services		1	19	0
Ontario Regional				
Algoma Public Health Unit	0	0	2	2
Brant County Health Unit	0	0	1	3

Chatham-Kent Health Unit	1	0	3	0
Durham Region Health Department	0	0	2	0
Eastern Ontario Health Unit	0	0	5	2
Grey Bruce Health Unit	0	0	2	2
Haliburton, Kawartha, Pine Ridge District Health Unit	0	0	5	0
Hamilton Public Health Services [3]	0	0	1	0
Hastings and Prince Edward Counties Health Unit	0	0	1	0
Huron Perth County Health Unit	0	0	1	1
Kingston, Frontenac and Lennox & Addington Health Unit	0	0	0	1
Lambton Health Unit	0	0	3	0
Leeds, Grenville and Lanark District Health Unit	0	0	2	0
Middlesex-London Health Unit	0	0	5	2
Niagara Region Public Health Department [4]	0	0	5	2
North Bay Parry Sound District Health Unit	0	0	0	1
Northwestern Health Unit	0	0	3	9
Ottawa Public Health	3	1	17	6
Peel Public Health [5]	0	0	1	1
Peterborough Public Health	0	0	4	0
Public Health Sudbury & Districts	0	0	0	1
Simcoe Muskoka District Health Unit	0	0	1	1
Thunder Bay District Health Unit	1	0	6	7
Toronto Public Health	1	0	11	2
Wellington-Dufferin-Guelph Health Unit	0	0	3	1
York Region Public Health Services [6]	0	0	4	1

[1] All social media accounts hosted by the Government of Manitoba

[2] The embedded Social media links were for Gov of PEI, not for PHE. It has separate twitter account for health which was not embedded

[3] All social media accounts hosted by the city of Hamilton

[4] All social media accounts hosted by the city of Niagara

[5] All social media accounts hosted by the Region of Peel

[6] All social media accounts hosted by the regional municipality of York

The table only includes the frequency of the PHEs that has at least one post in any category. The following PHEs did not have any social media posts with PD messaging and are not included in the table:

- Ontario regional: Haldimand-Norfolk Health Unit, Halton Region Health Department, Porcupine Health Unit, Region of Waterloo, Public Health, Renfrew County and District Health Unit, Southwestern Public Health, Windsor-Essex County Health Unit, Timiskaming Health Unit.

- Provincial/Territorial: Deena Hinshaw - The chief medical officer of Alberta, British Columbia Ministry of Health, Brent Roussin - The chief medical officer of Manitoba, Newfoundland and Labrador - Department of Health and community services, Janice Fitzgerald - The chief medical officer of Newfoundland and Labrador, Nova Scotia Department of Health and Wellness, Robert Strang - The chief medical officer of Nova Scotia, Nova Scotia Health Authority, Nunavut Department of Health and Social Services, Public Health Ontario, Health PEI, Horacio Arruda - The chief medical officer of Quebec, Saskatchewan Ministry of Health (Saskatchewan Health).
