# Supplementary material for: Physical distancing messages targeting youth on the social media accounts of Canadian public health entities and the use of behavioral change techniques
Source: BMC Public Health. 2021 Sep 7;21:1634. doi: 10.1186/s12889-021-11659-y (PMC8422061; doi:10.1186/s12889-021-11659-y)
Supplement: Supplementary file 6 — Additional file 6. Definitions and examples of BCTs found in social media posts with youth-targeted PD messages. Provides definitions of BCTs that were identified from the social media posts and examples in context of PD messages. [file 12889_2021_11659_MOESM6_ESM.pdf]

ADDITIONAL FILE 6: Definitions and examples of BCTs found in social media posts with youth-targeted PD messages

BCT	Definition*	Example in Physical Distancing context	Link to the post
Goals and Planning			
Goal setting (behaviour)	Set or agree on a goal defined in terms of physical distancing	If you're planning to keep active by going outside for a walk or a run, remember to keep at least 2 metres (6 feet) from others at all times. Avoid high-touch surfaces (handrails, doorknobs, etc.). Wash your hands well before leaving & as soon as you get back inside. www.hpph.ca/coronavirus	https://www.facebook.com/HuronPerthPublicHealth/photos/a.10153309689263530/10158346499308530/?type=1&theater
Action planning	Prompt detailed planning of practicing physical distancing (must include at least one of context, frequency, duration and intensity).	Easter social distancing<br>The long weekend is coming up. We know Easter and Passover are times of gathering for many, but it's important that we all maintain physical distancing this year. This means:<br>❌Do not gather with those who live outside of your household, either indoors or outside<br>❌Do not go for multiple grocery trips (make a list with your Easter dinner essentials and include it in your once-per-week grocery trip)<br>▯ Stay home when possible<br>▯ Celebrate virtually with family over video apps like Skype or Messenger (this includes celebrations like dinner or prayers)<br>▯ Host a virtual easter egg hunt<br>▯ Stay safe and enjoy yourselves!<br><br>While it is hard to not be able to get together to celebrate, we encourage you to stay socially connected while physically distanced.<br><br>We're #InThisTogether!	https://www.facebook.com/SaskHealthAuthority/videos/155123232488655/
Social support			

Social support (unspecified)	Advise on, arrange or provide social support or noncontingent praise or reward for practicing physical distancing. It includes encouragement and counselling, but only when it is directed at the behavior.	We know that physical distancing can make celebrations a bit more tough but let's embrace it and make it a celebration that won't be forgotten! Whether it's a birthday party, Easter, Ramadan, Passover, or any celebration with loved ones, make it a virtual party! You can still dine, laugh and play games together, even if you're apart. And if you live with others, take advantage of the company and spend some quality time together celebrating. There will be no celebration like it, so make it count and remember to #stayhome! .<br><br>#Socialdistance #physicaldistancing #untiltomorrow #socialdistancing #stayhomestaysafe #socialdistancingnow #socialdistancingclub #COVID19 #COVID #Coronavirus #COVID2019 #Canada #Health #community #love #getthefacts #publichealth #untiltomorrowchallenge #togetherathome #togetherbutapart #staycation #ournation #staysafe #together #happyeaster #easter #passover #ramadan #celebration	https://www.instagram.com/p/B-0k13Klkix/
Social support (emotional)	Advise on, arrange, or provide emotional social support for practicing physical distancing	Stay connected while staying in:<br>☆Video chat with friends, family, and colleagues using FaceTime, Skype, or Zoom<br>☆Connect through social media (but limit your daily screen time)<br>☆It's important and safe to talk about your feelings, open up to others, and reach out to friends and family to chat during this time<br>.<br>#Socialdistance #physicaldistancing #untiltomorrow #socialdistancing #stayhomestaysafe #socialdistancingnow #socialdistancingclub #COVID19 #COVID #Coronavirus #COVID2019 #Canada #CovidCanada #Health #Stayhealthy #stayhome #community #love #getthefacts #publichealth #wecandothis #untiltomorrowchallenge #togetherathome #togetherbutapart #togetherapart #stopthespread #family #friends #connected	https://www.instagram.com/p/B_BjJTeDb7x/

Instruction on how to perform the behavior	Advise or agree on how to perform the behavior	To accommodate physical distancing, people must continue to maintain a minimum distance of 2 metres between themselves and others at all times, with the exception of members of the same household or ‘bubble’. IN THE PICTURE: Keep 2m between you and others outside of your bubble, approximately the length of a bathtub, couch or moose antlers	https://www.facebook.com/GovNB/photos/a.222615324567875/1811858785643513/
Natural Consequences			
Information about health consequences	Provide information (e.g. written, verbal, visual) about health consequences of practicing physical distancing. Consequences can be for any target, not just the recipient(s) of the intervention	Ramadan is a month of self-reflection, gatherings within the community, daily prayer services, and self-discipline, including fasting daily from sunrise to sunset.<br><br>During this unprecedented time, it is encouraged that those observing Ramadan continue to practice #physicaldistancing throughout this month. This will help #flattenthecurve and keep you and your loved ones safe and healthy.<br><br>Do not have dinner gatherings with those outside your household and consider watching online prayer services from home.<br><br>Learn more about physical distancing: http://ow.ly/hisa50zhoxk	https://www.facebook.com/HealthyCdns/photos/a.10150334648756719/10158372722271719/
Information about social and environmental consequences	Provide information about social and environmental consequences of performing the behavior	Physical distancing is critical for protecting each other and our communities from COVID-19. On May 4th, and every day, be sure to stay 2 lightsaber lengths apart from others when outside your home. #MayThe4thBeWithYou #DoYourPartBC	https://www.facebook.com/photo?fbid=2870124106369922&set=a.1168855139830169
Comparison of Behaviour			
Demonstration of the behaviour	Provide an observable example of the maintaining physical distancing, directly in person or indirectly e.g. via film, pictures, for the person to aspire to or imitate	When practicing #PhysicalDistancing, you're allowed to chat w/your neighbour. Here's a sample of how that could go.<br>You: Hello.<br>Neighbour: Howdy, would you like to see this cute kitten video on my phone?<br>You: Sorry, Stu, I can't. We need to stay 2m away.	https://twitter.com/ottawahealth/status/1253286438009016320?s=20
Associations			

Prompts	Introduce or define environmental or social stimulus with the purpose of prompting or cueing the maintenance of physical distancing.	Today physical distancing circles were painted on the grass in #TrinityBellwoods Park as a pilot project to encourage people to help stop the spread of #COVID-19. Today physical distancing circles were painted on the grass in #TrinityBellwoods Park as a pilot project to encourage people to help stop the spread of #COVID-19. The circles, about 10-12 ft apart and 8 ft in diameter, are large enough for two or three people to sit or recline, but everyone should be from the same household. If the park is crowded and if no circles are available, people should visit a different park or come back later.<br><br>The paint used to create the circles is environmentally-friendly athletic field marking paint and is designed for active use and the longevity of the paint depends on grass growth, volume of foot traffic and proper drying time.<br><br>The circles will be assessed in 7-10 days when the grass will need to be cut. If the pilot is effective and successful in helping people to maintain physical distancing while at the park, we'll add paint circles in other popular destination or high-traffic parks.<br><br>You are encouraged to go out, get exercise and enjoy some time outside, but you must stay 2 metres (6 feet) apart from anyone who is not from your household. If physical distancing is not possible in a park, please leave to stay safe. To learn more about the rules in Toronto parks, go to toronto.ca/covid19, click 'Changes to City Services' and search for parks.	https://www.instagram.com/p/CAvfdnhhOKu/
Repetition and substitution			

Behavior substitution	Prompt substitution of the unwanted behavior with a wanted or neutral behavior	Social distancing does not have to mean social isolation. Connect with friends and family online.<br>.<br>This can include:<br>♣ Game nights □ Cooking parties □ Coffee breaks □ Tea time □ Birthday parties □ □ Group chats □ □ □ Exercise classes □ Art classes □ Music lessons □ Distance education □ □ Connect with old friends 🍽 Dinner parties 🕵 Scavenger hunts □ Dance parties □ Book clubs 📽 Movie nights □ Teddy bear picnics<br>.<br>For more information: durham.ca/mentalhealth<br>For COVID-19 updates: durham.ca/novelcoronavirus (link in bio)<br>#COVID19 #Coronavirus #FlattenTheCurve #PlankTheCurve #StayHome #Social Distancing #PhysicalDistancing #ProtectTheVulnerable #PublicHealthProtects #weareinthistogether	https://www.instagram.com/p/B-rmFLvJFmS/
Comparison of Outcomes			
Credible sources	Present verbal or visual communication from a credible source in favour of or against practicing physical distancing	"We are encouraging people to not use alone and either buddy up or access their local overdose prevention services" - Dr. Jane Buxton of the BCCDC. Overdose deaths increased 61% from February to March. Stay 2 metres from buddies to avoid passing the virus. http://ow.ly/O7e250zA0zR	https://twitter.com/PHSAofBC/status/1258460378406813697
Reward and Threat			
Future Punishment	Inform that future punishment or removal of reward will be a consequence of performance of an unwanted behavior	Stay Home, Toronto! The COVID-19 enforcement team will be patrolling parks again this weekend and ticketing those that do not practise physical distancing. Any 2 people who don't live together, who fail to keep 2 metres apart in a park, or those using closed park amenities, are subject to fines.<br><br>toronto.ca/COVID19<br><br>#COVID19 #coronavirus #Toronto #YYZ #stayhome #physicaldistancing	https://www.instagram.com/p/B_Iaa-2BmEy/
Regulation			

Reduce negative emotions	Advise on ways of reducing negative emotions to facilitate physical distancing, including stress management	Feeling overwhelmed and anxious about #COVID19? You are not alone. Here are some ways to take care of your mental health when #socialdistancing and self-isolating: http://ow.ly/vqsr50yXMHx #MentalHealth	https://twitter.com/PHSAofBC/status/1245801959971577856
Antecedents			
Restructuring the physical environment	Change, or advise to change the physical environment in order to facilitate performance of the wanted behavior or create barriers to the unwanted behavior	Tomorrow marks the start of Easter weekend. Traditionally, those celebrating may attend mass and gather with friends and family. This Easter, remember to practice #physicaldistancing and to consider watching Easter mass online instead. Don’t host events (inside or outside) like meals and parties for people who do not already live in your home. This will help limit the spread of #COVID19 within your community.<br><br>Learn more about physical distancing: http://ow.ly/l5Ra50z9pii #stayhomestaysafe	https://www.facebook.com/HealthyCdns/photos/a.10150334648756719/10158260257846719/
Restructuring the social environment	Change, or advise to change the social environment in order to facilitate practicing physical distancing or create barriers to the unwanted behavior	We all love our Moms SO MUCH... unfortunately this year that means celebrating at a distance if our moms aren't in the same household.<br>Here are a few alternate ways to celebrate the moms in your life!<br>• Call or video chat with all of your favourite mothers<br>• Host a virtual brunch with your extended family – you can even pre-cook and deliver* the meal<br>• Make gifts and cards at home and then send them or deliver*<br>• Catalogue your family’s favourite home cooked meals and get your mom to share her recipes and secret tips during a family video chat<br>• Create a Special Mother’s Day Slideshow or home video<br>*contactless delivery	https://www.instagram.com/p/B_-RiK1FjI9/

Avoidance/reducing exposure to cues for the behavior	Advise on how to avoid exposure to specific social and contextual/physical cues for practicing physical distancing	We know that physical distancing can make celebrations a bit more tough but let's embrace it and make it a celebration that won't be forgotten! Whether it's a birthday party, Easter, Ramadan, Passover, or any celebration with loved ones, make it a virtual party! You can still dine, laugh and play games together, even if you're apart. And if you live with others, take advantage of the company and spend some quality time together celebrating. There will be no celebration like it, so make it count and remember to #stayhome! .	https://www.instagram.com/p/B-0k13Klkix/
Distraction	Advise on how to avoid exposure to specific social and contextual/physical cues for the behavior, including changing daily or weekly routines	Got a case of the bores? Switch it up and try something new! Try your hand at painting, crack open a new book, learn a foreign language, start a journal and record your thoughts, watch a classic movie you've never seen or find out if you have a green thumb in the garden! . . . . . #Socialdistance #physicaldistancing #untiltomorrow #socialdistancing #stayhomestaysafe #isolation #quarantine #socialdistancingnow #covid19 #COVID #Coronavirus #COVID2019 #Canada #CovidCanada #Health #Stayhealthy #community #getthefacts #publichealth #untiltomorrowchallenge #holiday #longweekend #victoriaday #togetherathome #togetherapart #stopthespread #strongertogether #Canadian #flattenthecurve	https://www.instagram.com/p/CAWWeBTjfKY/
Identity			
Framing	Suggest the deliberate adoption of a perspective or new perspective on physical distancing (e.g. its purpose) in order to change cognitions or emotions about maintaining physical distancing	Physical distancing is not always possible for everyone. Some people need additional supports that require close contact. Continue to practice physical distancing to protect those who can't.<br><br>Learn more at https://bit.ly/PhysicalDistance-LPH #WeAreInThisTogether #PhysicalDistance #COVID19	https://twitter.com/lambton_ph/status/1267108898152108033

* BCT definition were adopted in physical distancing context from the original BCT definitions (1).

References:

Michie, S., Richardson, M., Johnston, M., Abraham, C., Francis, J., Hardeman, W., Wood, C. E. (2013). The behavior change technique taxonomy (v1) of 93 hierarchically clustered techniques: building an international consensus for the reporting of behavior change interventions. Ann Behav Med, 46(1), 81-95. doi: 10.1007/s12160-013-9486-6
